# The Ancient Office of Coroner

**Published:** 1911-05-06

**Authors:** F. J. Waldo

**Affiliations:** of the Middle Temple, Barrister-at-Law, H.M. Coroner for the City of London and Borough of Southwark.


### SPECIAL ARTICLE.

### THE ANCIENT OFFICE OF CORONER.

By F. J. WALDO, M.A., M.D. Camb., J.P., of the Middle Temple, Barrister-at-Law, H.M. Coroner for the City of London and Borough of Southwark.

(Abstract of a Lecture given before the Medico-Legal Society.)

The origin of the judicial office of the Coroner is lost in obscurity. In all probability it arose by a process of evolution from the earlier and more elementary Courts of Law. Some writers—among them Sir John Jervis, the leading authority on Coroners' law—have gone wide of the mark in their historical views. They assert that Coroners were in existence in the time of the Anglo-Saxon kings, Alfred and Athelstan. These statements are supported by reference to Nathaniel Bacon's "Historical and Political Discourse of the Laws and Governments of England" and to Dugdale's "Monasticum Anglicanum." Lord Coke made the same error, probably having been misled by Andrew Horn's "Mirror of Justices," a plausible but untrustworthy treatise of Anglo-Saxon laws written during the reign of Edward I. Bacon's work and Dugdale's "Monasticum" are now known to be equally unreliable.

### The First Coroner.

The first undoubted reference to the institution of Coroner occurs in a record setting forth a form of proceedings used at an "iter" or eyre or circuit of the King's Justices in September 1194. In Article 20, which provides for the election of three knights and a clerk as custodians of the pleas of the Crown, we find "*Preterea in quolibet comitatu eligantur tres milites et unus clericus custodes placitorum coronæ.*" There is, however, some evidence that the office had existed from the year 1100. It was not until the reign of Henry I. that the Coroner was required to try pleas of the Crown as well as keep the records.

The real object of the Coroner in those days was not so much to administer justice as to guard what may be called chance revenues falling to the King, such as forfeited chattels of felons, deodands, wrecks, royal fish, and treasure-trove, and much less diligence was often manifested to bring a felon to justice than to secure his chattels for the King. The inquisition in cases of persons dying suddenly or by violence was held chiefly to learn whether any property escheated to the Crown. The Coroner also received the confession and abjuration of felons who had fled to sanctuary; and, according to Bracton, held inquests into cases of rape and of arson. In the "Mirror of Justices" occurs the statement: "Coroners were wont also to hold their views in cases of sodomy, and on infant monsters who had nothing of humanity, or who had more of the beast than the man in them; and these the Coroner caused to be buried. But the holy faith grows stronger every day, whereby folk do not burden their souls with such horrible sins so commonly as they used."

### Functions of the Coroner.

On the passing of Magna Charta in 1215 provision was made, as one of the safeguards against the usurpation of royal authority "that no Sheriff, Constable, Coroner, or other of our Bailiffs shall hold pleas of our Crown." Notwithstanding this statute Coroners still continued to pass judgments on felons caught in the act, and, according to Britton and others, they also conducted jury trials in the County Court with or without the Sheriff in ordinary civil pleas.

Four of the functions of the mediæval Coroner survive at the present day; but inquiries into treasure-trove, outlawry, and acting for the Sheriff are rarely exercised.

The basis of modern practice is to be found in the famous statute "*De Officio Coronatoris*" of 1276. This statute enacted that the Coroner should go to the place where any person was slain or suddenly dead, and should by his warrant to the bailiffs or constables summon a jury from the four or five neighbouring towns to make inquiry on oath upon view of the body; and the Coroner and jury should inquire into the manner of killing and all circumstances that occasioned the party's death; who were present; whether the dead person was known; and where he lay the night before. They should examine the body to see if there were any signs of strangling about the neck, or of cords about the members, or of burns. If any appeared guilty of the murder the Coroner should inquire what goods, corn, and land he had, and then the dead body should be buried. Then followed regulations concerning "deodands"—that is to say, the things or chattels that caused death by misadventure—and were forfeited to the King to be applied to pious uses as a gift to God. Originally such gifts were actually bestowed on the Church for the good of the deceased's soul, or in the shape of alms. The chattel itself was not given up, but only its value as appraised by the jury, and paid for by the owner; and the Sheriffs were answerable for the amount. As pointed out by Dr. Sharpe, "the English term for deodand was 'bane'"—*i.e.* "the slayer" from the Anglo-Saxon "bana." We learn from mediæval records that deodands were frequently collected on account of fatal falls, such as from horses, boats, windows, ladders, or stairs often placed on the outside of the house in the mediæval city of London. From what we know of the habits of these times the Coroners would probably have been nearer the mark if, instead of taxing the objects named, they had gone back to the ultimate cause and laid the deodand on strong ale. It is worthy of note that the words in the statute "suddenly dead" apparently are not meant to include sudden deaths from fever, apoplexy, or other such visitations of God, but apply only to deaths from violence or unnatural causes.

Judging from the accounts given in the Coroners' rolls, deaths of persons in prison were frequent. During a period of one year and four months between 1322 and 1323, as a result of inquests held upon eleven male prisoners who died in the prison of the Castle of Northampton, as many verdicts of death from hunger, thirst, cold, and privation were returned. In one of these instances the three city Coroners sat together, and the jury brought in a verdict of "natural death from hunger and thirst, and not from the infliction of any other punishment."

In London, deodands went not to the King, but into the city coffers, although it is not clear what claim they had to such forfeitures. Deodands were abolished by statute in 1846 as "unreasonable and inconvenient."

### The Present-day Coroner.

The Coroner is a direct officer of the Crown. He is chosen for life or during good behaviour, and his office does not come to an end with the death of the Sovereign. As a King's officer he is responsible alone to the Lord Chancellor as representing the King. Down to 1888, county coroners, as in the earliest days, continued to be elected by the freeholders of the county much in the same way as in the election of members of Parliament. They are now elected in a less direct and popular way by the County Council on the old writ *de coronatore eligendo* still issued by the Lord Chancellor, and directed to the County Council instead of to the Sheriff. The old method of direct popular election entailed great cost upon candidates. Three elections for Central Middlesex before the year 1888 are said on good authority to have cost the successful candidates £12,000, £6,000, and £3,000 respectively.

### The Coroner's Jury.

In the thirteenth century the Coroner's jury as a rule consisted of thirty-two persons, twelve from the old parish governing body known as "the hundred," and five each from the four neighbouring townships. At the present day the Coroner issues his warrant for summoning not fewer than twelve nor more than twenty-three "good and lawful men." Those exempted from serving upon any inquests whatever, are, speaking broadly, all Peers, ministers of any religious denomination, those concerned in the administration of the law, members of the legal and medical professions, all Army and Navy officers on full pay, officers of the Post Office, Customs, Police, and other civil services.

### Some Suggestions.

The report presented by the Departmental Committee of the Home Office appointed in 1908 to inquire into the law relating to Coroners and Coroners' inquests contains a number of suggestions for amending the law. As regards qualification, the Committee advise that in future no one should be appointed a Coroner who is not a barrister, solicitor, or medical man. Another suggestion is to the effect that Coroners should be given power to order and pay for post-mortem examinations without an inquest necessarily following. But the committee is silent as to who is to make the examination, and as to whether the report of the pathologist is to be made in open court or upon oath, or in precisely what manner. Many suggestions are made on the contentious question of deaths under anæsthetics, among others to the effect that every death under an anæsthetic should be reported, and that the Coroner should be given discretionary power with regard to the holding or not of inquests in such cases. My own view is that if the cause of death is so doubtful after preliminary inquiry as to render a post-mortem necessary, that is a sufficient reason for the holding of an inquest.

